# The burden of rare variants in *DPYS* gene is a novel predictor of the risk of developing severe fluoropyrimidine-related toxicity

**DOI:** 10.1186/s40246-023-00546-9

**Published:** 2023-11-09

**Authors:** Elena De Mattia, Jerry Polesel, Marco Silvestri, Rossana Roncato, Lucia Scarabel, Stefano Calza, Michele Spina, Fabio Puglisi, Giuseppe Toffoli, Erika Cecchin

**Affiliations:** 1 Experimental and Clinical Pharmacology, Centro di Riferimento Oncologico di Aviano (CRO) IRCCS, Via Franco Gallini n. 2, 33081 Aviano, PN Italy; 2 Unit of Cancer Epidemiology, Centro Di Riferimento Oncologico Di Aviano (CRO) IRCCS, Via Franco Gallini n. 2, 33081 Aviano, PN Italy; 3 Department of Applied Research and Technological Development, Fondazione IRCCS Istituto Nazionale dei Tumori di Milano, Via Giacomo Venezian 1, 20133 Milan, Italy; 4 Department of Molecular and Translational Medicine, University of Brescia, Viale Europa 11, 25123 Brescia, Italy; 5 Department of Medical Oncology, Centro di Riferimento Oncologico di Aviano (CRO), IRCSS, Via Franco Gallini n. 2, 33081 Aviano, PN Italy; 6 Department of Medicine, University of Udine, Via Delle Scienze, 206, 33100 Udine, UD Italy

**Keywords:** DPYS, Rare variant, Fluoropyrimidine, Toxicity, Next-generation sequencing, Clinical implementation, PPARD

## Abstract

**Background:**

Despite a growing number of publications highlighting the potential impact on the therapy outcome, rare genetic variants (minor allele frequency < 1%) in genes associated to drug adsorption, distribution, metabolism, and elimination are poorly studied. Previously, rare germline *DPYD* missense variants were shown to identify a subset of fluoropyrimidine-treated patients at high risk for severe toxicity. Here, we investigate the impact of rare genetic variants in a panel of 54 other fluoropyrimidine-related genes on the risk of severe toxicity.

**Methods:**

The coding sequence and untranslated regions of 54 genes related to fluoropyrimidine pharmacokinetics/pharmacodynamics were analyzed by next-generation sequencing in 120 patients developing grade 3–5 toxicity (NCI-CTC vs3.0) and 104 matched controls. Sequence Kernel Association Test (SKAT) analysis was used to select genes with a burden of genetic variants significantly associated with risk of severe toxicity. The statistical association of common and rare genetic variants in selected genes was further investigated. The functional impact of genetic variants was assessed using two different in silico prediction tools (Predict2SNP; ADME Prediction Framework).

**Results:**

SKAT analysis highlighted *DPYS* and *PPARD* as genes with a genetic mutational burden significantly associated with risk of severe fluoropyrimidine-related toxicity (Bonferroni adjusted *P* = 0.024 and *P* = 0.039, respectively). Looking more closely at allele frequency, the burden of rare *DPYS* variants was significantly higher in patients with toxicity compared with controls (*P* = 0.047, Mann–Whitney test). Carrying at least one rare *DPYS* variant was associated with an approximately fourfold higher risk of severe cumulative (OR = 4.08, *P* = 0.030) and acute (OR = 4.21,* P* = 0.082) toxicity. The burden of *PPARD* rare genetic variants was not significantly related to toxicity. Some common variants with predictive value in *DPYS* and *PPARD* were also identified: *DPYS* rs143004875-T and *PPARD* rs2016520-T variants predicted an increased risk of severe cumulative (*P* = 0.002 and *P* = 0.001, respectively) and acute (*P* = 0.005 and* P* = 0.0001, respectively) toxicity.

**Conclusion:**

This work demonstrated that the rare mutational burden of *DPYS*, a gene strictly cooperating with *DPYD* in the catabolic pathway of fluoropyrimidines, is a promising pharmacogenetic marker for precision dosing of fluoropyrimidines. Additionally, some common genetic polymorphisms in *DPYS* and *PPARD* were identified as promising predictive markers that warrant further investigation.

**Supplementary Information:**

The online version contains supplementary material available at 10.1186/s40246-023-00546-9.

## Introduction

Human germline rare (minor allelic frequency [MAF] < 1%) and novel variants are increasingly being investigated for their promising pharmacogenetic value as predictive markers of pharmacological outcome of therapy [1–5]. Through integrated analysis of data from the 1000 Genomes Project and the Exome Sequencing Project, it was estimated that approximately 30–40% of the total functional variability in pharmacogenes (i.e., transporters, phase I and II enzymes, nuclear receptors) is caused by rare variants that are generally not captured by standard targeted genotyping tests [6]. On this basis, it has been hypothesized that rare genetic variants should account for a substantial portion of the unexplained inter-individual variability in drug metabolism phenotypes, pharmacokinetics, and adverse drug reaction (ADR) risk, especially for certain drugs (e.g., warfarin, irinotecan) [7]. Some confirmatory data come from a few clinical studies that found a significant association between the burden of genetic variants with low minor allele frequency in certain pharmacogenes and the occurrence of adverse drug reactions [3] or a poor efficacy outcome [5] after pharmacological treatment.

We have previously shown that the burden of rare variants in the dihydropyrimidine dehydrogenase (*DPYD,* DPD*)* gene is significantly increased in patients experiencing severe toxicity to fluoropyrimidines (i.e., 5-fluorouracil [5-FU] and its oral prodrug capecitabine) [2]. Severe toxicity to fluoropyrimidines remains an emerging concern in oncology, with up to 30% of patients experiencing severe toxicity, which can be fatal in approximately 1% of patients [8, 9]. Although a pre-treatment *DPYD* testing increases the safety of treatment with fluoropyrimidines and is now recommended by the European Medicines Agency [10], the currently validated four-variant panel (i.e., DPYD*2A, rs3918290; DPYD*13, rs55886062; c.2846A > T, rs67376798; c.1236G > A-HapB3; rs56038477) leaves many toxic events potentially related to patient genetic characteristics unexplained [11]. In this context, testing rare and novel missense *DPYD* variants may collectively capture an appreciable additional fraction of patients at increased risk for severe fluoropyrimidine-related toxicity [2].

However, the metabolic pathway of fluoropyrimidines is quite complex, and in addition to DPD, several other enzymes and metabolic proteins are involved in determining drug bioavailability and exposure [12]. Several previous studies have explored the potential role of genetic polymorphisms in genes such as thymidylate synthase (TYMS) or methylenetetrahydrofolate reductase (MTHFR) [12]. The results were encouraging for specific polymorphisms, even though no conclusive agreement has yet been reached on the clinical validity of these markers. However, the potential impact of assessing the global or gene-specific burden of rare genetic variants in genes involved in the fluoropyrimidine pathway as a predictive marker for the risk of developing severe toxicity has, to our knowledge, never been evaluated.

The primary aim of the present work was to test the role of rare and novel variants in an expanded set of fluoropyrimidine-related genes in predicting the risk of severe fluoropyrimidine-related toxicity. To this end, we assessed the genetic sequence of a panel of 54 fluoropyrimidine-related genes using a next-generation sequencing (NGS)-based method in a clinically characterized group of 120 patients who experienced severe fluoropyrimidine-related toxicity and 104 matched control subjects.

## Patients and methods

### Patient cohorts and clinical data collection

The cases (“toxicity group”) and controls (“no-toxicity group”) included in the study were selected from a biobank of clinical cases prospectively collected at the Clinical and Experimental Pharmacology Unit of the Centro di Riferimento Oncologico (CRO), IRCCS, in Aviano (PN), Italy [13]. The selection criteria have been published previously [2]. Briefly, patients in the “toxicity group” (*n* = 120) met the following criteria: (1) histologically confirmed diagnosis of solid cancer; (2) available peripheral biological blood sample; (3) available detailed clinical data (4); treatment with fluoropyrimidines (5-FU or capecitabine); (5) absence of any acknowledged *DPYD* risk genetic variant (i.e., DPYD*2A, DPYD*13, c.2846A > T, c.1236G > A-HapB3); and (6) development of at least one episode of hematologic or non-hematologic fluoropyrimidine-related toxicity of grade ≥ 3 according to the Common Terminology Criteria for Adverse Events (CTCAE) v.3.0. Patients in the “no-toxicity group” (*n* = 104) were selected according to the same criteria as above, with the exception of item (6), and were not allowed to be statistically different overall from the “toxicity group” with respect to key clinical–demographic characteristics (e.g., sex, age, tumor type, setting of treatment, chemotherapy regimen).

The method for retrieving clinical and toxicity data from patients’ medical records was already described [13]. The maximum grade of toxicity experienced by the patients during the entire chemotherapy course was considered for patients’ selection.

All patients in the study were self-reported Caucasian. The study protocol complied with the ethical guidelines of the 1975 Declaration of Helsinki. The protocol was approved by the Comitato Etico Indipendente-Centro di Riferimento Oncologico di Aviano. All patients provided written informed consent for genetic analysis before entering the study. All experiments were carried out in accordance with the relevant guidelines and regulations of Centro di Riferimento Oncologico di Aviano.

### Candidate genes and polymorphism selection

Candidate genes were selected based on a literature search (PubMed-MEDLINE) focusing on those encoding proteins involved in fluoropyrimidine-related pathways (e.g., drug metabolism, folate pathway, drug membrane transporters, gene transcriptional regulators, epigenetic control). In the end, a panel of 54 genes was selected and included in the pharmacogenetic analysis. The custom design of the panel was performed using NimbleDesign software based on Genome Build hg19/GRCh37 (February 2009) and captured the genetic variability of all exons and their adjacent splice junctions (approximately 35 bases upstream and downstream of the exon) as well as the 5’ and 3’ untranslated regions (UTRs). For two genes of particular relevance for fluoropyrimidine-response pathway (*TYMS*, *MTHFR*), an additional 3000 bp was added in 5′ of the first exon of each gene to include potential proximal promoters. The list of genes included in the panel (*n* = 54), the subpathway (*n* = 6) to which they belong, and the number of bases covered by the design for each gene are shown in Additional file 1: Table S1. The sequencing data of the *DPYD* gene have been previously analyzed and published [2]; therefore, the *DPYD* gene is not included in the present work.

### Library preparation and sequencing

Genomic DNA was extracted from peripheral blood samples using the BioRobot EZ1 automated extractor in association with the “EZ1 DNA Blood Kit 350μl” kit (Qiagen) and stored at + 4 °C. To improve the quality of the extracted DNA, DNA purification was also performed using Agencourt AMPure XP beads (Beckman Coulter). The quality (i.e., 260/280 and 260/230 ratio) and quantity of DNA samples were assessed using the Nanodrop spectrophotometric method (Thermo Scientific) and the Quantus™ Fluorometer (Promega), respectively.

Gene sequencing was performed using a custom hybrid capture-based Roche/NimbleGen assay. DNA library preparation for all samples was conducted on 100 ng of input purified DNA. Library preparation was performed according to the manufacturer’s instructions in the SeqCap EZ HyperCap Workflow User’s Guide v 2.3 (Roche). Briefly, genomic DNA was enzymatically fragmented and linked to the index; after adapter ligation, double-sided selection was performed to obtain fragments of approximately 300–350bp, which were then amplified. Each library was pooled before in-solution hybridization to a custom NimbleGen SeqCap EZ Choice Library (Roche) of complementary oligonucleotide DNA baits. After washing, the captured libraries are amplified by PCR. The concentration of single and pooled libraries was evaluated by Quantus™ Fluorometer (Promega), while the quality and size distribution were assessed by 2200 TapeStation system (Agilent). Pooled libraries were sequenced on a Miseq platform (Illumina), according to the manufacturer’s instructions, using a 300-cycle kit with 2 × 150 paired-end read setup. NGS sequencing data were validated by Sanger sequencing with a 100% of concordance [2].

### Bioinformatic analysis, variants annotation, and functional prediction

Raw sequencing data were quality checked using FastQC [14] and aligned to Human reference genome (hg19/GRCh37) using BWA-MEM software [15]. Alignment sequencing data were quality checked using Qualimap [16] and removed for duplicated sequences using Picard [17]. Bedtools2 [18] was used to compute mapped reads for each genomic position specified in the manifest file of the panel. Mutect2 from GATK4.1 was employed for calling germline variants and Oncotator for variant annotation. To reduce the number of false-positive calls and obtain a list of confident genetic variations, variants with at least one of the following features were screened out: (1) Variant Classification = “nontranslated intergenic regions (IGR)” or “intron”; (2) read position = “FAIL”; (3) bad haplotype = “FAIL”; (4) base quality = “FAIL”; (5) mapping quality = “FAIL”; (6) strand artifact = “FAIL”; (7) clustered events = “FAIL”; (8) fragment_length = ”FAIL”; (9) t_lod = ”FAIL”; (10) multiallelic = ”FAIIL”; (11) read depth < 50X; (12) variant allele frequency < 0.15 to reduce the risk of false positive calls; (13) variants in genomic regions outside the panel. Unsupervised hierarchical clustering with Ward.D2 linkage and Euclidean distance was used to identify patient groups characterized by low coverage over the gene panel. Moreover, all genes defined by a median coverage < 50 in at least 95% of the entire cohort were excluded from downstream analysis.

MAF from the 1000 Genomes database (European population) was used to classify germline variants into very rare (MAF ≤ 0.1%), rare (0.1% < MAF < 1%), common (MAF ≥ 1%), and novel (MAF not registered; absence of rs ID in dbSNP database [19]. Only in case of no data available in 1000 Genomes European population database, ExAC database (non-Finnish European population) was considered.

The PredictSNP algorithm [20] was used to predict the functional impact of the identified variants by classifying them into deleterious and tolerated. For the missense variants, an alternative method based on absorption, distribution, metabolism, and excretion (ADME)-optimized prediction framework (APF) of Zhou et al. [21] was used. Particularly, seven in silico functional prediction algorithms from Ensembl’s variant effect predictor (VEP) [22] were used (i.e., SIFT, Sorting Intolerant From Tolerant; Polyphen-2, Polymorphism Phenotyping v2; LRT, Likelihood Ratio Test; MutationAssessor; PROVEAN, Protein Variation Effect Analyzer; VEST3, Variant Effect Scoring Tool; CADD, Combined Annotation-Dependent Depletion) with the threshold of deleteriousness optimized by APF. To identify variants predicted to be deleterious, the prediction score (PS) of each variant's was calculated as *N*_del_ / *N*_tot_, where *N*_tot_ is the total number of algorithms available for predictions and *N*_del_ is the number of algorithms that predicted the variant to be deleterious. The missense variants with PS ≥ 0.5 were classified as “deleterious.”

### Statistical analysis

Socio-demographic and clinical characteristics were reported as proportions, and differences between toxicity and no-toxicity groups were assessed using Fisher’s exact test.

**Fig. 1:**
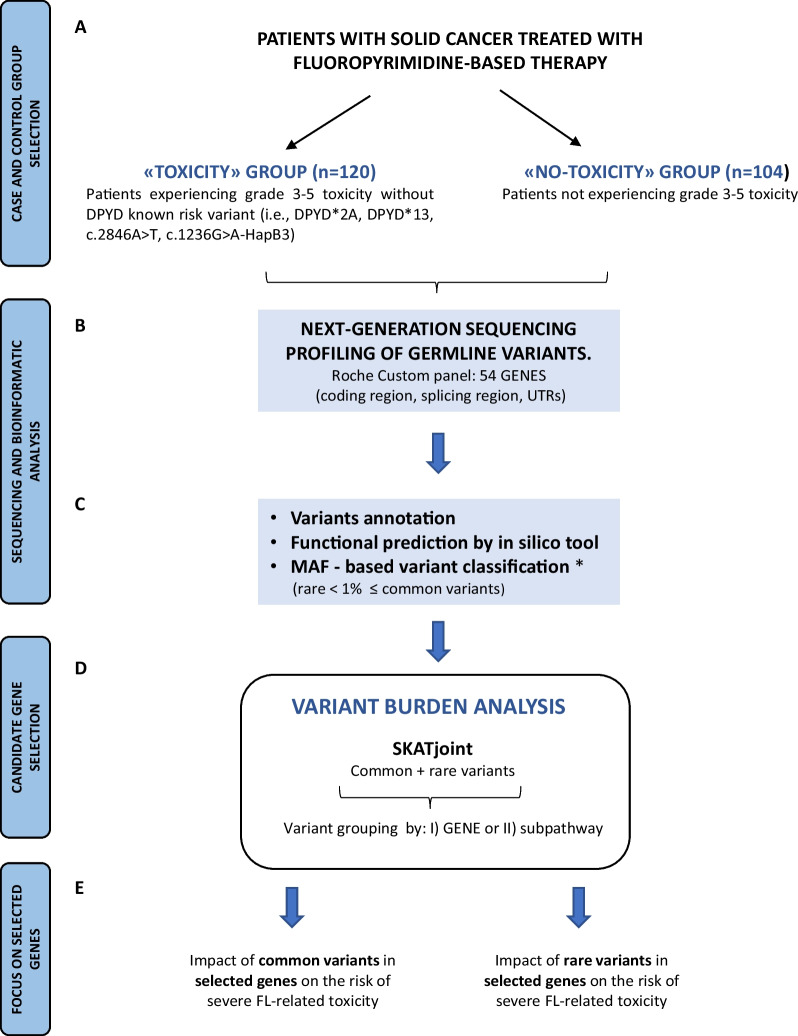
Flowchart of the study. **a** The study population included a total of 224 patients with solid cancers treated with fluoropyrimidine (FL)-based therapy divided into cases (“toxicity group”) and controls (“no-toxicity group”). **b** Blood-derived DNA from cases and controls was genotyped using a customized targeted next-generation sequencing panel comprising 54 genes. **c** Raw sequencing data were processed through a data analysis pipeline that allowed identification/annotation of germline genetic variants. Variant classification based on minor allele frequency (MAF, common ≥ 1% and rare < 1%) and functional annotation of variants were also performed. **d** Variant analysis was performed using the Sequence Kernel Association Test (SKAT) for common and rare variants combined (SKATjoint). Variants were grouped at the gene and subpathway levels. These analyses allowed identification of candidate genes associated with risk of severe FL-related toxicity. **e** The predictive role of common or rare variants in the selected candidate genes for the risk of developing severe FL-related toxicity was further investigated. *MAF-based variant classification: very rare, MAF ≤ 0.1%; rare, 0.1% < MAF < 1%; common, MAF ≥ 1%; novel, MAF not registered + no rs ID. In SKAT analysis, very rare and novel variants are included in the rare group

The flowchart of the study is illustrated in Fig. 1. The association between variants and toxicity was evaluated through the Sequence Kernel Association Test (SKAT) [23]. Due to the small sample size, it was not possible to evaluate only rare variants, so common and rare variants were combined in the screening phase to select candidate genes/pathways. The R package SKATjoint was used. The algorithm SKAT was applied at two levels of grouping: a) at the gene level, where all variants in a gene region were combined; b) at the pathway level, where all variants in genes of the same pathway were combined. For each gene or pathway, the *P-*value was further adjusted according to the Bonferroni correction taking into account the number of groups tested within each level of grouping.

For genes and pathways that emerged from SKAT screening, the number of rare variants was counted for each patient, distinguishing by variant type and MAF frequency (i.e., < 1%, < 0.1%, novel). For each group, the mean number of variants was calculated as the ratio between the total number of variants and the number of patients. Considering the low frequency, the mean number of variants was expressed per 100 patients by multiplying it by 100; differences between groups were evaluated through Mann–Whitney test. For common variants, the genotype for each polymorphism was reported, and differences between the two study groups were evaluated through the Fisher’s exact test.

Toxicity risk was assessed by odds ratio (OR) and corresponding 95% confidence intervals (CI) estimated using an unconditional logistic regression model, adjusting for sex, age, cancer site, treatment setting, and fluoropyrimidines. Statistical significance was claimed for *p* < 0.05 (two-sided). The sample size was determined according to the criterion of feasibility by including all patients available in our biobank who met the inclusion criteria. Statistical analyses were performed using SAS System 9.4 and R 4.1.

## Results

### Sequencing and patient characteristics

A total of 224 fluoropyrimidine-treated patients (*n* = 120 in the “toxicity group”; *n* = 104 in the “no-toxicity group”) were sequenced by NGS. After quality control, 11 of the 224 sequenced samples were excluded from analysis because they did not pass the quality check of sequencing data by fastQC. All of these samples were characterized by a low number of produced paired-end reads (number of reads < 1,000,000) and a sequence length > 220 bp versus the expected 150–200 bp, indicating a failure in the sequencing step. An additional 35 samples were excluded from analysis based on the result of unsupervised cluster analysis, which classified the sample as “good” or “poor” based on coverage. Thus, the final “toxicity group” included 82 patients, whereas the “no-toxicity group” included 96 patients. Nine genes (i.e., *UPRT, TYMP, RXRA, TK1, MIR27B, MIR27A, MIR23A, KDM6A, KDM6B*) were excluded from the analysis because of unfavorable coverage (i.e., not meeting the criteria of median depth coverage of 50X for the genes defined by the panel in at least 95% of the samples).

Overall, the coverage analysis showed a median number of 542,162 mapped reads (range: 61,074–1563800) with a median percentage of reads at 1 × and 10x (depth of coverage) of 86% and 2%, respectively. Looking at coverage at the genomic level, the median depth at the gene level was homogeneous between the “toxicity” and “no-toxicity” groups, allowing comparison of the two cohorts.

The main clinical–demographic features of the two study populations are shown in Table 1. The two groups were balanced overall in terms of clinical–demographic characteristics, with the exception of treatment setting, as the adjuvant setting was more frequent among the controls while first-line or further lines of treatment were more frequent among the cases. Of the 82 patients in the “toxicity group,” 50 (61.0%) developed grade 3 toxicity and 32 (39.0%) developed grade 4 toxicity as the maximum grade of hematologic or non-hematologic toxicity experienced during the entire course of treatment. Forty-seven of 82 patients (57.3%) developed grade ≥ 3 hematologic toxicity, with neutropenia being the most common adverse event (35/47; 74.5%). Fifty-eight of 82 patients (70.7%) developed grade ≥ 3 non-hematologic toxicity, with diarrhea being the most common adverse event (27/58; 46.6%). Thirty-five of 82 patients (42.7%) experienced severe hematologic or non-hematologic toxicity within the first cycle of treatment (acute toxicity, cycle ≤ 1), and forty-seven patients (57.3%) after the first cycle.

**Table 1:** Socio-demographic and clinical characteristic of solid cancer patients enrolled in the study

	Toxicity group (*n* = 82)		No-toxicity group (*n* = 96)		Fisher’s exact test (*P*)
	*n*	(%)	*n*	(%)	
Sex					
Female	46	(56.1)	46	(47.9)	
Male	36	(43.9)	50	(52.1)	0.295
Age, years (median, range)	62	(52–68)	64	(51–68)	0.511
Cancer type					
Colon	49	(59.8)	66	(68.8)	
Rectum	14	(17.1)	17	(17.7)	
Breast	5	(6.1)	5	(5.2)	
Stomach	4	(4.9)	1	(1.0)	
Head and neck	1	(1.2)	3	(3.1)	
Pancreas	1	(1.2)	3	(3.1)	
Others	2	(2.4)	1	(1.0)	
Unknown	6	(7.3)	0	(0.0)	0.078
Chemotherapy					
Fluoropyrimidines					
*5-Fluorouracil*	68	(82.9)	81	(84.4)	
*Capecitabine*	14	(17.1)	15	(15.6)	0.840
Monotherapy	8	(9.8)	7	(7.3)	
Association with oxaliplatin	18	(22.0)	38	(39.6)	
Association with irinotecan	38	(46.3)	35	(36.5)	
Association with other drugs	18	(22.0)	16	(16.7)	0.091
Therapy setting					
Neo-adjuvant	5	(6.1)	8	(8.3)	
Adjuvant	22	(26.8)	42	(43.8)	
First-line or further lines	55	(67.1)	46	(47.9)	0.034
Max toxicity grade*					
3	50	(61.0)	–		
4	32	(39.0)	–		

*Maximum grade of hematological or non-hematological toxicity experienced by the patients

### Variants identified

A total of 7,420 and 7,896 germline variants were called against the reference genome in the “toxicity” and “no-toxicity” groups, respectively. The mean coverage (read depth) of the identified genetic variants was 97 (range: 50–530) and 75 (range: 50–214) for the “toxicity” and “no-toxicity” groups, respectively.

**Fig. 2:**
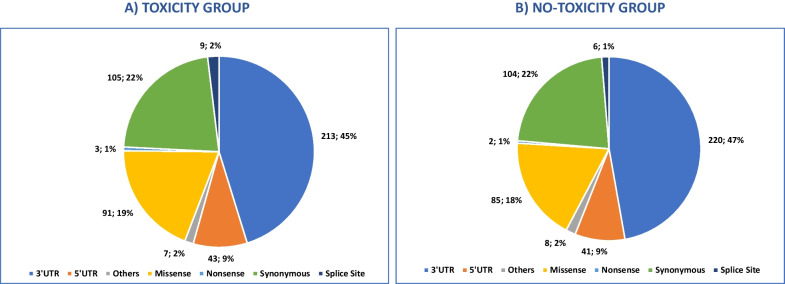
Pie chart visualizing the type of genetic germline variants identified in groups. **A** “toxicity” and **B** “no-toxicity” groups. “Other” variants include: De novo Start OutOfFrame, Frame Shift Del, Frame Shift Ins, In Frame Ins, and Start Codon SNP

A total of 471 unique genetic variants were identified (426 single-nucleotide polymorphisms [SNP], 25 deletions [DEL], 17 insertions [INS], 3 double-nucleotide polymorphisms [DNP]) in the “toxicity group” (Fig. 2A). Of these, 275/471 (58.4%) were common (MAF ≥ 1%) and 196/471 (41.6%) were rare/very rare (MAF < 1%) or novel variants. Thirty-six of the 275 (13.1%) common variants and 45 of the 196 (23.0%) rare/very rare or novel variants were predicted as deleterious. Most of the identified variants were in the 3’ region (213/471, 45.2%). Of the remaining variants, 105/471 (22.3%) were synonymous, 91/471 (19.3%) were missense and 43/471 (9.1%) were in the 5’ region.

In the “no-toxicity” group, 466 unique genetic variants were identified (414 SNP, 31 DEL, 17 INS, 3 DNP, 1 oligonucleotide polymorphism [ONP]). Of these 274/466 (58.8%) were common (MAF ≥ 1%) and 192/466 (41.2%) were rare/very rare (MAF < 1%) or novel variants. Thirty-five of the 274 (12.8%) common variants and 44 of the 192 (22.9%) rare/very rare or novel variants were classified as deleterious. Most of the identified variants were in the 3’ region (220/466, 47.2%). Of the remaining variants, 104/466 (22.3%) were synonymous variants, 85/466 (18.2%) were missense variants and 41/466 (8.8%) were in the 5’ region (Fig. 2B).

**Fig. 3:**
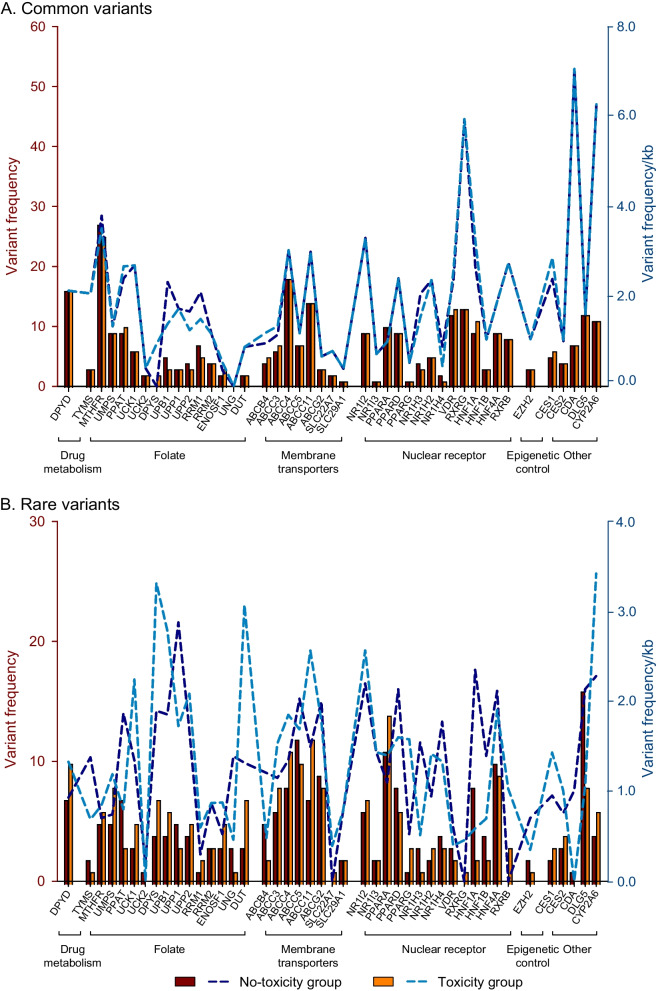
Frequency of common (MAF ≥ 1%) and rare (MAF < 1%, including novel) variants according to gene in patients with and without toxicity

**Fig. 4:**
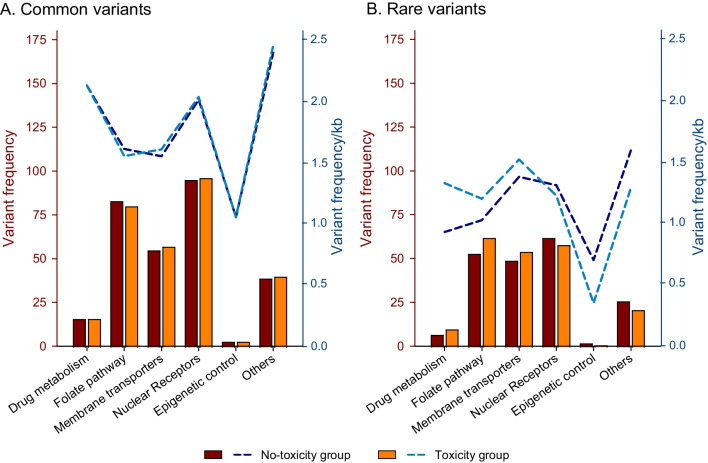
Frequency of common (MAF ≥ 1%) and rare (MAF < 1%, including novel) variants according to subpathway in patients with and without toxicity

The frequencies of common and rare (including novel) variants by gene and subpathway in the “toxicity” and “no-toxicity” groups are shown in Figs. 3 and 4, respectively. As can be seen from the figures, the frequency distribution of the common variants in the two groups is similar at both at gene and subpathway level, while the frequency distribution of the rare variants in the two groups is remarkably different.

### Variants burden analysis

The role of rare/novel and common variants and pathways in the risk of developing severe fluoropyrimidine-related toxicity was assessed by SKATjoint analysis. The list of genes and related subpathways is reported in Additional file 1: Table S1. The SKAT statistics were calculated considering all variants, variants in the UTRs, and missense variants, grouping them also for the functional effect. The results of the SKATs analysis are shown in Table 2.

**Table 2:** Gene and pathway-level SKAT output

Common and rare variants (SKATjoint)^a^			
Gene (HGNC)	crude.*P*	BH.*P*	Bonf.*P*
*All variants*			
*ABCB4*	0.041	0.196	1
*ABCC3*	0.020	0.136	0.882
*ABCC4*	0.053	0.196	1
*ABCC5*	0.021	0.136	0.943
*CYP2A6*	0.010	0.136	0.432
***DPYS***	**0.001**	**0.024**	**0.024**
*NR1I3*	0.049	0.196	1
*PPARD*	0.022	0.136	0.954
*SLC22A7*	0.015	0.136	0.645
*VDR*	0.003	0.066	0.131
*DLG5*	0.052	0.196	1
*RRM2*	0.044	0.196	1
*All variants deleterious*			
*ABCB4*	0.0168	0.3019	0.604
*NR1H3*	0.0336	0.4036	1
***PPARD***	**0.0011**	**0.0389**	**0.039**
*VDR*	0.0497	0.4475	1
*3’UTR/5’UTR*			
*ABCC5*	0.030	0.304	1
*CES1*	0.023	0.304	0.925
*DPYS*	0.002	0.072	0.072
*MTHFR*	0.045	0.366	1
*VDR*	0.018	0.304	0.754
*3’UTR/5’UTR deleterious*			
***PPARD***	**0.001**	**0.022**	**0.022**
*Missense*			
*ABCB4*	0.010	0.306	0.376
*CYP2A6*	0.051	0.306	1
*ENOSF1*	0.045	0.306	1
*NR1H3*	0.048	0.306	1
*RRM2*	0.018	0.306	0.645
*VDR*	0.050	0.306	1
*Missense deleterious*			
*ABCB4*	0.017	0.278	0.520
*NR1H3*	0.048	0.385	1
*RRM2*	0.018	0.278	0.555
*VDR*	0.050	0.385	1

Common and rare variants (SKATjoint)^a^			
Subpathway	crude.*P*	BH.*P*	Bonf.*P*
*All variants*			
**Nuclear Receptor**	**0.004**	**0.018**	**0.033**
**Membrane transporter**	**0.004**	**0.018**	**0.037**
*All variants deleterious*			
Nuclear receptor	0.008	0.075	0.075
*3’UTR/5’UTR*			
Nuclear receptor	0.049	0.212	0.444
*3’UTR/5’UTR deleterious*			
Nuclear receptor	0.016	0.095	0.095
*Missense*			
Nuclear receptor	0.044	0.229	0.395
*Missense deleterious*			
–			

Only the associations with crude *P* < 0.05 are reported. Association with also Bonferroni *P* < 0.05 is evidenced in bold

^a^*P* values were estimated adjusting for sex, age, cancer site, treatment setting, and fluoropyrimidines

The variants are classified as common (MAF ≥ 1%) and rare (MAF < 1%). Novel variants (MAF not registered; absence of rs ID in dbSNP database) are included in the rare

The *DPYS* (all variants, *P* = 0.024) and the *PPARD* (all variants deleterious, *P* = 0.039; 3’UTR/5’UTR variants deleterious, *P* = 0.022) genes were identified to significantly impact the risk of developing fluoropyrimidine-related toxicity by applying the Bonferroni correction. At subpathway level, “nuclear receptor” (all variants, *p* = 0.033) and “membrane transporter” (all variants, *P* = 0.037) resulted the classes of genes significantly associated with the risk of severe toxicity.

### Rare variants and risk of toxicity

Based on the results of the SKAT analysis (Table 2), the role of the burden of rare and novel variants in the *DPYS* and *PPARD* genes on the risk of severe fluoropyrimidine-related toxicity was further investigated. The complete list of rare (MAF < 1%) and novel variants identified in the *DPYS* and *PPARD* genes in the “toxicity” and “no-toxicity” groups is shown in the Additional file 1: Tables S2 and S3, respectively.

As for the *DPYS* gene, it was significant in the “all variants” group: seven and four rare/very rare variants were detected in the case and control groups, respectively (Additional file 1: Table S2). The mean number of *DPYS* variants per 100 patients was significantly higher in the “toxicity” group that in the “no-toxicity” group (*P* = 0.047). The number of patients with at least one rare *DPYS* variant differed between groups (12.2% and 4.2% in the “toxicity” and “no-toxicity” groups, respectively), albeit with borderline significance (*P* = 0.055). Carrying at least one rare *DPYS* variant was associated with an approximately fourfold higher risk of severe cumulative toxicity (*P* = 0.030) after accounting for potential confounders in the multivariable model (Table 3). The same effect was detected for acute severe toxicity, although not significantly (*P* = 0.082). It was confirmed that the presence of at least one rare *DPYD* missense variant in the present study population increased the risk of cumulative severe toxicity, as previously published [2], although not significantly due to the small sample size (OR = 6.03; 95% CI 0.65–56.27; *P* = 0.115). After excluding five cases and one control with rare *DPYD* missense mutations from the analysis, the rare *DPYS* variants maintained their negative impact on the risk of severe cumulative toxicity (OR = 4.06; 95% CI 1.12–14.81; *P* = 0.115).

**Table 3:** Germline burden of rare (including novel) genetic variants and the risk of developing severe fluoropyrimidine-related toxicity

Gene	Mean number of mutations per 100 patients*			Patients with mutations					OR (95% CI)^a^	*P* value	Cycle ≤ 1	
	Toxicity		M-W test (*P*)	Toxicity				Fisher’s exact test (*P*)			OR (95% CI)^a^	*P*
	No	Yes		No		Yes						
				*n*	(%)	*n*	(%)					
*All variants*												
*DPYS*	4.2	13.4	0.047	4	(4.2)	10	(12.2)	0.055	4.08 (1.15–14.49)	0.030	4.21 (0.83–21.33)	0.082
*All variants deleterious*												
*PPARD*	1.0	2.4	0.472	1	(1.0)	2	(2.4)	0.595	1.48 (0.12–17.73)	0.759	1.54 (0.08–31.18)	0.777

The risk of acute toxicity (cycle ≤ 1) was also considered

^a^Odds ratio (OR) and corresponding 95% confidence intervals (CI) were estimated from multinomial regression model, adjusting for sex, age, cancer site, treatment setting, and fluoropyrimidines

* (Number of genetic variants/total number of patients)*100

The *PPARD* gene was significantly associated with toxicity in both “all variants deleterious” and “3’UTR/5’UTR variants deleterious” groups; the two groups overlapped because all deleterious variants identified were in the UTR region (Additional file 1: Table S3). The mean number of *PPARD* variants per 100 patients was not significantly higher in the “toxicity” group than in the “no-toxicity” group (*P* = 0.472). The number of patients with at least one rare *PPARD* variant was not significantly different between groups (*P* = 0.595). Carrying at least one rare *PPARD* variant was not associated with the risk of experiencing severe cumulative (*P* = 0.759) or acute (*P* = 0.777) severe toxicity in multivariate analysis (Table 3).

### Common variants and risk of toxicity

The role of *DPYS* and *PPARD* polymorphisms (MAF > 1%) as predictors of severe toxicity was further investigated. The toxicity risk for each common *DPYS* (all variants) and *PPARD* (deleterious 3’UTR/5’UTR variants) variant identified by NGS analysis was compared between the “toxicity” and “no-toxicity” groups and is summarized in Table 4.

**Table 4:** Risk of toxicity according to common polymorphisms of *DPYS* (all variants) and *PPARD* (variants in the 3’UTR/5’UTR)

	Cumulative toxicity					Acute toxicity (Cycle ≤ 1)				
	Yes (*n* = 82)		No (*n* = 96)		OR (95% CI)^a^	Yes (*n* = 37)		No (*n* = 96)		OR (95% CI)^a^
	*n*	(%)	*n*	(%)		*n*	(%)	*n*	(%)	
*DPYS*-rs2298840										
GG	80	(97.6)	96	(100)	Ref	36	(97.3)	96	(100)	Ref
GA	2	(2.4)	0	(0.0)	–	1	(2.7)	0	(0.0)	–
	Fisher’s exact test: *P* = 0.211					Fisher’s exact test: *P* = 0.278				
*DPYS*-rs143004875										
-/-	74	(90.2)	96	(100)	Ref	33	(89.9)	96	(100)	Ref
-/T	8	(9.8)	0	(0.0)	–	4	(10.8)	0	(0.0)	–
	Fisher’s exact test: ***P* = 0.002**					Fisher’s exact test: ***P* = 0.005**				
*PPARD*-rs2016520										
CC	28	(34.2)	59	(61.5)	Ref	8	(21.6)	59	(61.5)	Ref
CT	22	(26.8)	18	(18.8)	2.60 (1.14–5.87)	14	(37.8)	18	(18.8)	6.54 (2.16–19.80)
TT	32	(39.0)	19	(19.8)	3.54 (1.66–7.56)	15	(40.5)	19	(19.8)	5.96 (2.06–17.20)
	Fisher’s exact test: ***P* = 0.001**					Fisher’s exact test: ***P* = 0.0001**				
*PPARD*-rs9658170										
GG	80	(97.6)	93	(96.9)	Ref	36	(97.3)	93	(96.9)	Ref
GA	2	(2.4)	3	(3.1)	0.52 (0.07–4.09)	1	(2.7)	3	(3.1)	0.37 (0.03–5.05)
	Fisher’s exact test: *P* = 1.000					Fisher’s exact test: *P* = 1.000				
*PPARD*-rs9658167										
GG	79	(96.3)	93	(96.9)	Ref	35	(94.6)	93	(96.9)	Ref
GA	3	(3.7)	3	(3.1)	1.75 (0.32–9.43)	2	(5.4)	3	(3.1)	3.57 (0.49–26.19)
	Fisher’s exact test: *P* = 1.000					Fisher’s exact test: *P* = 0.618				

*P* value < 0.05 is evidenced in bold

^a^Odds ratio (OR) and corresponding 95% confidence intervals (CI) were estimated from multinomial regression model, adjusting for sex, age, cancer site, treatment setting, and fluoropyrimidines

Two common polymorphisms were detected in the *DPYS* gene, the synonymous rs2298840 (c.216C > T; *p*.Phe72Phe) and the 3’UTR rs143004875 (g.chr8:105391734_105391735insT). Remarkably, these variants were identified only in the “toxicity” group, whereas they were not detected in the “no-toxicity” group. Although statistical analysis is hampered by the small number of cases, the rs143004875-T variant allele was associated with an increased risk of severe toxicity for both cumulative (*P* = 0.002) and acute toxicity (*P* = 0.005). A similar trend was observed for rs2298840, with all patients carrying the polymorphic A-allele developing severe toxicity.

As for the *PPARD* gene, all three identified deleterious variants are located in the UTR region: rs2016520 (5’UTR, g.chr6:35378778C > T), rs9658170 (3’UTR, g.chr6:35394504G > A), and rs9658167 (3’UTR, g.chr6:35394080G > A). Among these variants, rs2016520 was associated with the risk of developing severe cumulative (*P* = 0.001) and acute toxicity (*P* = 0.0001). Particularly, patients with the rs2016520-TT genotype had about threefold and sixfold higher risk of severe cumulative and acute toxicity, respectively.

To investigate in detail the functional role of the *DPYS*-rs2298840, *DPYS*-rs143004875, and *PPARD*-rs2016520 polymorphisms, bioinformatic analysis was performed using HaploReg v4.1 [24], RegulomeDB v2.0.3 [25], and Ensembl’s VEP-Ensembl GRCh37release release 110—July 2023 [22]. The methods and detailed results are summarized in Additional file 1: Table S4. In brief, the synonymous *DPYS*-rs2298840 variant could have a moderate impact on gene functionality and/or expression, as it broadly alters regulatory chromatin status and consensus sequence for transcription factors and exhibits eQTL hits. An impact on the splicing mechanism could also not be excluded. All these effects were summarized by a RegulomeDB rank score of 3a (i.e., transcription factors binding + any motif + DNase peak) and a probability score of 0.61235. The CADD score is 14.91. A similar functional prediction was obtained for the 3’UTR *DPYS*-rs143004875 polymorphism, although the effects appear to be smaller (RegulomeDB rank score = 6 [i.e., motif hit], probability score = 0.22365; CADD score not available). The 5’UTR *PPARD*-rs2016520 variant, that is in linkage (*r*^2^ ≥ 0.8) with 17 additional polymorphisms, could have a moderate functional consequence since it affects chromatin architecture and DNA accessibility for gene transcription, it is located in a transcriptional binding element with a resulting impact on the regulation of protein expression (eQTL hits) and regulates the splicing mechanism. All of these effects were summarized by a RegulomeDB rank score of 5 (i.e., transcription factors binding or DNase peak) and a probability score of 0.13454. The CADD score is 16.81.

## Discussion

The burden of rare germline variants in pharmacogenes appears to be a promising marker for personalization of pharmacological treatment. Specific fields of pharmacotherapy, such as the use of fluoropyrimidines in cancer patients, are severely affected by the occurrence of adverse drug reactions, and currently used pharmacogenetic markers, such as *DPYD* genetic variants, are very effective in reducing the risk of toxicity in carriers but leave many toxicity cases unexplained.

In this study, we explored the predictive effect of rare variants burden in genes other than *DPYD* on the occurrence of severe toxicity to fluoropyrimidines. The main finding was the identification, for the first time, of the critical role of rare variant burden (MAF < 1%) in the *DPYS* gene as predictive a marker for the risk of severe fluoropyrimidine-related toxicity.

Dihydropyrimidinase *(DPYS,* DHPase), also known as 5,6-dihydropyrimidine amidohydrolase, is the second enzyme in the 3-step catabolic pathway of fluoropyrimidines downstream of the action of DPD. In vitro studies have shown that alterations in DHPase activity modulate the clearance of 5-FU and that decreased DHPase functionality is associated with increased sensitivity to 5-FU [26]. Moreover, *DPYS* genetic variants have been found to contribute to inter-individual predisposition to the development of severe fluoropyrimidine-related toxicity, particularly in Asian populations [27–31]

This work is the first to report that the burden of *DPYS* rare variants, including novel variants, identified by NGS, is associated with the risk of fluoropyrimidine-related toxicity. Carriers of rare *DPYS* variants had an approximately a fourfold increased risk of developing severe to fatal fluoropyrimidine-related toxicities in the first treatment cycle as well as during the entire course of chemotherapy. It could be hypothesized that DHPase deficiency, possibly caused by rare variants that negatively affect enzyme activity, leads to accumulation of dihydrofluorouracil (FUH_2_), which can be converted back to 5-FU by the reversible reaction catalyzed by DPD, eventually leading to overexposure and toxicity [32]. Interestingly, in our study, the predictive value of the rare *DPYS* variants remained significant even when patients previously identified as carrying a rare *DPYD* missense associated with severe toxicity risk [2] were excluded from the analysis, suggesting an independent effect of the two pharmacogenetic markers. Thus, these data demonstrated the potential role of rare genetic *DPYS* variants in personalizing fluoropyrimidine-based therapy, especially in cancer patients with normal *DPYD* activity, and further research efforts are certainly needed to translate these findings into clinical practice.

In addition to the rare variants burden, the current study identified common polymorphisms in the *DPYS* gene as further promising markers of toxicity. Particularly, two common variants, the synonymous rs2298840 and the 3’UTR rs143004875, were detected only in the “toxicity” group and not in the “no-toxicity” group and could therefore be considered for the first time as potentially significant predictors of the risk of developing severe fluoropyrimidine-related toxicity. No functional data on the effects of the rs2298840 and rs143004875 polymorphisms on DHPase activity were found in the published literature. However, a preliminary in silico assessment suggested a potential moderate functional impact, particularly for the rs2298840 variant, which was largely associated with an alteration in regulatory chromatin states and the consensus sequence for transcription factors, ultimately potentially affecting gene expression.

As for common polymorphisms, promising data were also found for peroxisome proliferator-activated receptor delta (PPARD), a member of the nuclear receptors superfamily that includes transcription factors that play crucial roles regulating drug metabolism enzymes and membrane transporter genes [33–35]. A pharmacological role of PPARD has been suggested, and its activation has been shown to contribute to the modulation of the cytotoxic effects of antineoplastic drugs [35]. Interestingly, a recent gene expression analysis based on data from The Cancer Genome Atlas liver hepatocellular carcinoma project suggests the involvement of PPARD in the hepatic regulation of *DPYD* expression [36]. In the present study, the common *PPARD* polymorphism rs2016520 was found to be a predictor of severe fluoropyrimidine-related toxicity, with patients with the TT genotype showing an increased risk of developing adverse events. Data from literature suggest that this genetic variant may affect PPARD functionality by interfering with Sp-1 binding, resulting in higher transcriptional activity of the C allele compared with the T allele in vitro [37]. Therefore, it could be hypothesized that altered PPARD activity associated with the rs2016520 variant could alter the regulation of *DPYD* expression and thus the catabolism of fluoropyrimidines, ultimately affecting the risk of severe toxicity. Further larger studies are needed to confirm this suggestive hypothesis and the clinical relevance of the *PPARD* rs2016520 polymorphism.

At the subpathway level, the present study suggested that the burden of common and rare variants in the “nuclear receptor” and “membrane transporter” gene classes may play a role in predicting severe fluoropyrimidine-related toxicity by SKAT analysis. The potential crucial role of the burden of rare variant in membrane transporter genes as predictive markers of therapy outcome is confirmed by literature data. Rare damaging variants in the transporter OATP1B1 *(SLCO1B1)* have been shown to affect methotrexate clearance in children with acute lymphoblastic leukemia [4]. A recent study has reported that breast cancer patients with a high burden of rare variants in the transporter gene ABCC1 (MRP1) have shorter disease-specific survival than patients with a low variant burden after therapy with the MRP1 substrates cyclophosphamide and doxorubicin [5]. These results confirm the potential predictive value of rare variants in membrane transporter genes and call for further research efforts on this topic.

Some limitations of the present study need to be considered. First, the study included patients treated mainly with fluoropyrimidines in combination with other chemotherapeutic agent (i.e., irinotecan, oxaliplatin). While particular attention was paid to exclude severe toxicities clearly not related to fluoropyrimidines, possible interference cannot be excluded when recording toxicities not all attributable to fluoropyrimidines Second, the in silico prediction tools for synonymous variants as well as for variants falling into the untranslated or splice regions are still inadequate, which could have a negative impact on the assignment of deleteriousness of the same variants. Therefore, a more comprehensive functional analysis of the role of deleterious rare/novel variants in toxicity risk was not possible. Third, given the relatively small sample size of this work, confirmation of the results obtained in a larger population is needed, especially with regard to the predictive effect of common polymorphisms.

In conclusion, the present study demonstrated for the first time that the burden of rare variants in the *DPYS* gene detected by NGS can be a promising predictive marker for the risk of developing severe fluoropyrimidine-related toxicity. This finding contributed to identify additional factors predisposing to the occurrence of severe toxicity of chemotherapy with fluoropyrimidines besides *DPYD* variants and may improve treatment personalization, especially in cancer patients with normal DPD activity. A predictive role of common *DPYS* and *PPARD* polymorphisms was also highlighted and requires future studies to confirm the data presented here. The present work demonstrated that the clinical predictive value of the burden of rare germline variants in candidate genes is still unexplored but has great potential to revolutionize the future of pharmacogenetics.

### Supplementary Information

**Additional file 1: Table S1:** Candidate genes selected for pharmacogenetic analysis, the subpathway to which they belong, and the number of bases covered by the design for each gene. **Table S2:** Rare (MAF<1%) and very rare (MAF≤0.1%) variants identified in the DPYS gene (transcript ENST00000351513.7) in the (**A**) “toxicity” and (**B**) “no-toxicity” groups. **Table S3:** Rare (MAF<1%) and very rare (MAF≤0.1%) variants identified in the PPARD gene (transcript ENST00000360694.8) in the (**A**) “toxicity” and (**B**) “no-toxicity” groups. **Table S4:** In silico predicted functional effect of DPYS-rs2298840, DPYS-rs143004875 and PPARD-rs2016520 polymorphisms.

## Data Availability

The datasets generated and analyzed during the current study are not publicly available due to ethical restrictions but are available from the corresponding author on reasonable request.

## Abbreviations

5-FU: 5-Fluorouracil
ADME: Absorption, distribution, metabolism, and excretion
ADR: Adverse drug reaction
APF: ADME-optimized prediction framework
DEL: Deletions
DNP: Double-nucleotide polymorphisms
*DPYD (*DPD): Dihydropyrimidine dehydrogenase
*DPYS*: Dihydropyrimidinase
INS: Insertions
MAF: Minor allelic frequency
MTHFR: Methylenetetrahydrofolate reductase
NGS: Next-generation sequencing
ONP: Oligonucleotide polymorphism
PPARD: Proliferator-activated receptor delta
PS: Prediction score
SKAT: Sequence Kernel Association Test
SNP: Single-nucleotide polymorphisms
TYMS: Thymidylate synthase
UTRs: Untranslated regions
VEP: Variant effect predictor
