# Supplementary material for: The burden of rare variants in DPYS gene is a novel predictor of the risk of developing severe fluoropyrimidine-related toxicity
Source: Hum Genomics. 2023 Nov 9;17:99. doi: 10.1186/s40246-023-00546-9 (PMC10633914; doi:10.1186/s40246-023-00546-9)
Supplement: Supplementary file 1 — Additional file 1: Table S1: Candidate genes selected for pharmacogenetic analysis, the subpathway to which they belong, and the number of bases covered by the design for each gene. Table S2: Rare (MAF<1%) and very rare (MAF≤0.1%) variants identified in the DPYS gene (transcript ENST00000351513.7) in the (A) “toxicity” and (B) “no-toxicity” groups. Table S3: Rare (MAF<1%) and very rare (MAF≤0.1%) variants identified in the PPARD gene (transcript ENST00000360694.8) in the (A) “toxicity” and (B) “no-toxicity” groups. Table S4: In silico predicted functional effect of DPYS-rs2298840, DPYS-rs143004875 and PPARD-rs2016520 polymorphisms. [file 40246_2023_546_MOESM1_ESM.docx]

**Supplementary Table S1:** Candidate genes selected for pharmacogenetic analysis, the subpathway to which they belong, and the number of bases covered by the design for each gene.

| Gene | HGNC ID | Subpathway | Chromosome | bases covered |
| --- | --- | --- | --- | --- |
| *DPYD* | HGNC:3012 | Drug metabolism | chr1 | 7526 |
| *TYMS/TYMSOS* | HGNC:12441 / HGNC:29553 | Folate pathway | chr18 | 1452 |
| *MTHFR* | HGNC:7436 | Folate pathway | chr1 | 7120 |
| *UMPS* | HGNC:12563 | Folate pathway | chr3 | 6726 |
| *PPAT* | HGNC:9238 | Folate pathway | chr4 | 3747 |
| *UCK1* | HGNC:14859 | Folate pathway | chr9 | 2230 |
| *UCK2* | HGNC:12562 | Folate pathway | chr1 | 4964 |
| *DPYS* | HGNC:3013 | Folate pathway | chr8 | 2111 |
| *UPB1* | HGNC:16297 | Folate pathway | chr22 | 2157 |
| *UPP1* | HGNC:12576 | Folate pathway | chr7 | 1738 |
| *UPP2* | HGNC:23061 | Folate pathway | chr2 | 2400 |
| *TYMP* | HGNC:3148 | Folate pathway | chr22 | 1875 |
| *TK1* | HGNC:11830 | Folate pathway | chr17 | 1671 |
| *RRM1* | HGNC:10451 | Folate pathway | chr11 | 3341 |
| *RRM2* | HGNC:10452 | Folate pathway | chr2 | 3449 |
| *UPRT* | HGNC:28334 | Folate pathway | chrX | 2625 |
| *ENOSF1* | HGNC:30365 | Folate pathway | chr18 | 5715 |
| *UNG* | HGNC:12572 | Folate pathway | chr12 | 2158 |
| *DUT* | HGNC:3078 | Folate pathway | chr15 | 2278 |
| *ABCB4* | HGNC:45 | Membrane transporter | chr7 | 4152 |
| *ABCC3* | HGNC:54 | Membrane transporter | chr17 | 5220 |
| *ABCC4* | HGNC:55 | Membrane transporter | chr13 | 5946 |
| *ABCC5* | HGNC:56 | Membrane transporter | chr3 | 5916 |
| *ABCC11* | HGNC:14639 | Membrane transporter | chr16 | 4681 |
| *ABCG2* | HGNC:74 | Membrane transporter | chr4 | 4504 |
| *SLC22A7* | HGNC:10971 | Membrane transporter | chr6 | 2542 |
| *SLC29A1* | HGNC:11003 | Membrane transporter | chr6 | 2539 |
| *NR1I2* | HGNC:7968 | Nuclear Receptor | chr3 | 2729 |
| *NR1I3* | HGNC:7969 | Nuclear Receptor | chr1 | 1394 |
| *PPARA* | HGNC:9232 | Nuclear Receptor | chr22 | 10012 |
| *PPARD* | HGNC:9235 | Nuclear Receptor | chr6 | 3749 |
| *PPARG* | HGNC:9236 | Nuclear Receptor | chr3 | 1903 |
| *NR1H3* | HGNC:7966 | Nuclear Receptor | chr11 | 1945 |
| *NR1H2* | HGNC:7965 | Nuclear Receptor | chr19 | 2116 |
| *NR1H4* | HGNC:7967 | Nuclear Receptor | chr12 | 2256 |
| *VDR* | HGNC:12679 | Nuclear Receptor | chr12 | 5049 |
| *RXRA* | HGNC:10477 | Nuclear Receptor | chr9 | 5515 |
| *RXRB* | HGNC:10478 | Nuclear Receptor | chr6 | 2916 |
| *RXRG* | HGNC:10479 | Nuclear Receptor | chr1 | 2189 |
| *HNF1A* | HGNC:11621 | Nuclear Receptor | chr12 | 3405 |
| *HNF1B* | HGNC:11630 | Nuclear Receptor | chr17 | 2867 |
| *HNF4A* | HGNC:5024 | Nuclear Receptor | chr20 | 4728 |
| *MIR27A* | HGNC:31613 | Epigenetic contol | chr19 | 99 |
| *MIR27B* | HGNC:31614 | Epigenetic contol | chr9 | 99 |
| *MIR23A* | HGNC:31605 | Epigenetic contol | chr19 | 99 |
| *EZH2* | HGNC:3527 | Epigenetic contol | chr7 | 2850 |
| *KDM6A* | HGNC:12637 | Epigenetic contol | chrX | 6084 |
| *KDM6B* | HGNC:29012 | Epigenetic contol | chr17 | 6752 |
| *CES1* | HGNC:1863 | Others | chr16 | 2098 |
| *CES2* | HGNC:1864 | Others | chr16 | 3968 |
| *CDA* | HGNC:1712 | Others | chr1 | 992 |
| *DLG5* | HGNC:2904 | Others | chr10 | 7491 |
| *CYP2A6* | HGNC:2610 | Others | chr19 | 1754 |

**Supplementary Table S2: Rare (MAF<1%) and very rare (MAF≤0.1%) variants identified in the *DPYS* gene (transcript ENST00000351513.7) in the (A) “toxicity” and (B) “no-toxicity” groups.**

1. **TOXICITY GROUP**

| **Coordinates (GRCh37)** | **Type** | | **Rs ID** | **Ref Allele** | **Alt Allele** | **Exon** | **Codon Change** | **Protein Change** | **Freq*** | **Classification** | **Functional Prediction** | **Pts§** |
| --- | --- | --- | --- | --- | --- | --- | --- | --- | --- | --- | --- | --- |
| chr8:105391742 | SNP | 3'UTR | rs73699419 | A | T | 0 | NA | NA | 0.003 | Rare | TOLERATED | 2 |
| chr8:105391917 | DEL | 3'UTR | rs539754034 | AGAC | - | 0 | NA | NA | 0.002 | Rare | NA | 1 |
| chr8:105440236 | SNP | Missense | rs376965972 | C | T | 6 | c.(1063-1065)cGg>cAg | p.R355Q | < 0.0001 | Very Rare | DELETERIOUS | 1 |
| chr8:105440271 | SNP | Synonymous | rs117104587 | G | C | 6 | c.(1027-1029)acC>acG | p.T343T | 0.009 | Rare | TOLERATED | 2 |
| chr8:105456597 | SNP | Synonymous | rs7825427 | T | C | 4 | c.(670-672)gcA>gcG | p.A224A | 0.002 | Rare | TOLERATED | 1 |
| chr8:105459576 | SNP | Synonymous | rs188038278 | C | T | 3 | c.(577-579)gcG>gcA | p.A193A | < 0.0001 | Very Rare | TOLERATED | 1 |
| chr8:105459627 | SNP | Synonymous | rs143825702 | G | A | 3 | c.(526-528)taC>taT | p.Y176Y | 0.004 | Rare | TOLERATED | 3 |

1. **NO-TOXICITY GROUP**

| **Coordinates (GRCh37)** | **Type** | | **Rs ID** | **Ref Allele** | **Alt Allele** | **Exon** | **Codon Change** | **Protein Change** | **Freq*** | **Classification** | **Functional Prediction** | **Pts§** |
| --- | --- | --- | --- | --- | --- | --- | --- | --- | --- | --- | --- | --- |
| chr8:105391917 | DEL | 3'UTR | rs539754034 | AGAC | - | 0 | NA | NA | 0.002 | Rare | NA | 1 |
| chr8:105405065 | SNP | Missense | rs775971165 | G | A | 8 | c.(1390-1392)Cct>Tct | p.P464S | < 0.0001 | Very Rare | DELETERIOUS | 1 |
| chr8:105440238 | SNP | Synonymous | rs35013010 | A | G | 6 | c.(1060-1062)gaT>gaC | p.D354D | < 0.0001 | Very Rare | TOLERATED | 1 |
| chr8:105440273 | SNP | Missense | rs201457190 | T | C | 6 | c.(1027-1029)Acc>Gcc | p.T343A | 0.001 | Very Rare | DELETERIOUS | 1 |

* Frequency data obtained by Ensembl (<http://www.ensembl.org/index.html>) from 1000 Genomes European population database (EUR, European) or gnomADe genomes (Non-Finnish European) database. § Number of patients harboring the polymorphism. Abbreviations: DEL, deletion; na, not available; Pts, patients; SNP, single nucleotide polymorphism.

**Supplementary Table S3: Rare (MAF<1%) and very rare (MAF≤0.1%) variants identified in the *PPARD* gene (transcript ENST00000360694.8) in the (A) “toxicity” and (B) “no-toxicity” groups.**

1. **TOXICITY GROUP**

| **Coordinates (GRCh37)** | **Type** | | **Rs ID** | **Ref Allele** | **Alt Allele** | **Exon** | **Codon Change** | **Protein Change** | **Freq*** | **Classification** | **Functional Prediction** | **Pts§** |
| --- | --- | --- | --- | --- | --- | --- | --- | --- | --- | --- | --- | --- |
| chr6:35394947 | SNP | 3'UTR | rs9658176 | C | T | 0 | NA | NA | 0.009 | Rare | TOLERATED | 1 |
| chr6:35395568 | SNP | 3'UTR | rs62402066 | G | A | 0 | NA | NA | 0.003 | Rare | TOLERATED | 1 |
| chr6:35393636 | SNP | Missense | rs772150465 | G | A | 9 | c.(1105-1107)cGg>cAg | p.R369Q | < 0.0001 | Very Rare | TOLERATED | 1 |
| chr6:35378844 | SNP | 5'UTR | rs201971336 | G | T | 0 | NA | NA | 0.001 | Very Rare | DELETERIOUS | 2 |
| chr6:35395047 | DEL | 3'UTR | -- | CCTGCAGGCTCCATGCACCTCCCTTCCCTCCCTGAGGCAGGTGAGAACCCAGAGAGAGGGG | - | 0 | NA | NA | -- | Novel | NA | 1 |
| chr6:35378798 | SNP | 5'UTR | rs9658134 | G | A | 0 | NA | NA | 0.006 | Rare | TOLERATED | 2 |

1. **NO-TOXICITY GROUP**

| **Coordinates (GRCh37)** | **Type** | | **Rs ID** | **Ref Allele** | **Alt Allele** | **Exon** | **Codon Change** | **Protein Change** | **Freq*** | **Classification** | **Functional Prediction** | **Pts§** |
| --- | --- | --- | --- | --- | --- | --- | --- | --- | --- | --- | --- | --- |
| chr6:35394947 | SNP | 3'UTR | rs9658176 | C | T | 0 | NA | NA | 0.009 | Rare | TOLERATED | 2 |
| chr6:35395795 | SNP | 3'UTR | rs9794 | G | A | 0 | NA | NA | < 0,0001 | Rare | TOLERATED | 2 |
| chr6:35395761 | SNP | 3'UTR | rs200476846 | C | T | 0 | NA | NA | 0.002 | Rare | TOLERATED | 1 |
| chr6:35395864 | SNP | 3'UTR | -- | T | A | 0 | NA | NA | -- | Novel | DELETERIOUS | 1 |
| chr6:35395004 | SNP | 3'UTR | rs200077865 | G | A | 0 | NA | NA | < 0.0001 | Very Rare | TOLERATED | 1 |
| chr6:35378798 | SNP | 5'UTR | rs9658134 | G | A | 0 | NA | NA | 0.006 | Rare | TOLERATED | 2 |
| chr6:35388031 | SNP | Synonymous | rs765704308 | C | T | 5 | c.(256-258)taC>taT | p.Y86Y | < 0.0001 | Very Rare | TOLERATED | 1 |
| chr6:35395025 | SNP | 3'UTR | rs1057198325 | C | A | 0 | NA | NA | < 0.0001 | Very Rare | TOLERATED | 1 |
| chr6:35395448 | SNP | 3'UTR | rs9658177 | G | A | 0 | NA | NA | 0.001 | Very Rare | TOLERATED | 1 |

* Frequency data obtained by Ensembl (<http://www.ensembl.org/index.html>) from 1000 Genomes European population database (EUR, European) or gnomADe genomes (Non-Finnish European) database. § Number of patients harboring the polymorphism. Abbreviations: DEL, deletion; na, not available; Pts, patients; SNP, single nucleotide polymorphism.

**Supplementary Table S4:** *In silico* predicted functional effect of *DPYS-*rs2298840, *DPYS-*rs143004875 and *PPARD*-rs2016520 polymorphisms.

The functional prediction of the putative effect of candidate polymorphisms was performed using three online software: HaploReg v4.1 (<https://pubs.broadinstitute.org/mammals/haploreg/haploreg.php>; RegulomeDB v2.0.3 (<https://regulomedb.org/regulome-search/>) and Ensembl’s Variant Effect Predictor (VEP) Ensembl GRCh37release release 110 - July 2023 (https://grch37.ensembl.org/info/docs/tools/vep/index.html). Accessed on September 1, 2023. A stringency of r2=0.80 and 1000 Genomes Project (EUR) dataset were chosen for haploblock identification by HaploReg. Genome Build hg19/GRCh37 (Feb 2009) was used.

| **General data** | | | **HaploReg** | | | | | | **Ensembl’s VEP** | | | **RegulomeDB** | | |
| --- | --- | --- | --- | --- | --- | --- | --- | --- | --- | --- | --- | --- | --- | --- |
| **dbSNP ID** | **SNPs in the haploblock** | **Chromosome Location (GRCh37) and SNP Typology** | **Promoter histone marks** | **Enhancer**  **histone marks** | **DNAse** | **Motifs changed** | **GRASP QTL hits** | **Selected eQTL hits** | **CADD** | **Impact on Splicing** | **Feature Type** | **Rank^** | **Score^^** | **Peaks** |
| *DPYS*  rs2298840 | rs4602859; rs3750187 | chr8:105478933  (exon 1; Phe72Phe) | 13 tissues  (ESC, ESDR, IPSC, FAT, BRN, SKIN, VAS, LIV, GI, OVRY, PANC, HRT, THYM) | 10 tissues (BRST, BLD, GI, ADRL, BRN, HRT, MUS, LNG, PLCNT, SPLN) | 15 tissues (ESC,ESDR,IPSC,IPSC,BLD,BLD,SKIN,GI,KID,LNG,THYM,MUS,BLD,BRN,SKIN) | 5 altered motifs  (Irf,PRDM1,PU.1,SRF,p300) | -- | 3 hits | 14.91 | yes | -- | 3a | 0.61235 | 120 |
| *DPYS*  rs143004875 | -- | chr8:105391734  3’UTR | -- | 3 tissues (ESC, ESDR, IPSC) | -- | 3 altered motifs  (HDAC2,Mef2,TATA) | -- | -- | -- | -- | Enhancer | 6 | 0.22365 | 5 |
| *PPARD*  rs2016520 | rs7749165; rs1040436; rs9470015; rs2267665; rs1883322; rs2267666; rs7751481; rs2267667; rs2038068; rs2038067; rs1003973; rs2267668; rs2267669; rs6899536; rs200858190; rs2299871; rs2395622 | chr6:35378778  5’UTR | -- | 6 tissues (ESDR, IPSC, BLD, SKIN, PLCNT, THYM) | 1 tissue  (SKIN) | 2 altered motifs  (CTCF,Rad21) | 3 hits | 4 hits | 16.81 | yes | -- | 5 | 0.13454 | 8 |

^ Rank score, computed based on the integration of multiple high-throughput datasets, ranges from 1 to 7 with the lower value indicating the stronger evidence for a variant to be in a functional region. 3a= TF binding + any motif + DNase peak; 5= TF binding or DNase peak; 6= Motif hit

^^ Probability score ranges from 0 to 1, with 1 being most likely to be a regulatory variant.

Abbreviation: CADD, Combined Annotation Dependent Depletion, SNP, single nucleotide polymorphism; UTR, untranslated region.
